# Null association between *ACE* gene *I/D* polymorphism and diabetic nephropathy among multiethnic Malaysian subjects

**DOI:** 10.4103/0971-6866.69351

**Published:** 2010

**Authors:** Jaime J. Jayapalan, Sekaran Muniandy, Siew P. Chan

**Affiliations:** Department of Molecular Medicine, Faculty of Medicine, University of Malaya, Kuala Lumpur, Malaysia; 1 Department of Medicine, Faculty of Medicine, University of Malaya, Kuala Lumpur, Malaysia

**Keywords:** *ACE I/D* polymorphism, type 2 diabetes mellitus, diabetic nephropathy, Malaysian population

## Abstract

**BACKGROUND:**

Wide inter-ethnic allelic variations of the Angiotensin Converting Enzyme (*ACE*) i nsertion-deletion (I/D) gene polymorphism were thought to be responsible for the conflicting gene–diabetic nephropathy disease association worldwide. We have investigated the genetic susceptibility of the *ACE* gene to diabetic nephropathy in the multiethnic Malaysian population.

**MATERIALS AND METHODS:**

A total of 137 healthy (control) and 256 diabetic subjects were recruited. The diabetic subjects were further subdivided according to their nephropathy status based on urinary albumin-creatinine ratio (ACR) and glomerular filtration rate (GFR). Triple primer polymerase chain reaction (PCR) was used for *ACE* I/D genotyping. Subsequently, populationwide genetic analysis and gene-disease association studies were performed.

**RESULTS:**

The genotype frequencies in all subgroups were in Hardy-Weinberg equilibrium. Similar allelic and genotypic frequency of *ACE* I/D gene polymorphism was observed between healthy controls versus pooled type 2 diabetes mellitus (T2DM) subjects, and normoalbuminuria versus microalbuminuria, macroalbuminuria and End Stage Renal Failure (ESRF) (*P* > 0.05). Neither ethnicity nor gender exerted any influence on the *ACE* I/D gene polymorphism (*P* > 0.05), with the exception of the Chinese ethnic group which exhibited a higher frequency of ID genotype (*P* = 0.042). A multinomial logistic regression model showed that predictive factors including age, systolic blood pressure (SBP), high density lipoprotein (HDL) and glycosylated hemoglobin (HbA1C) were independently associated with diabetic nephropathy, in that order.

**CONCLUSION:**

The I/D polymorphism of the *ACE* gene is not significantly associated with both T2DM and/or diabetic nephropathy in this Malaysian population regardless of ethnicity and gender.

## Introduction

Diabetic nephropathy is a syndrome characterized by albuminuria in which the urinary albumin excretion rate rises progressively over time leading to declining glomerular filtration rate (GFR) and renal fibrosis with loss of renal function, coupled with arterial hypertension, increased cardiovascular disease risk and eventually end stage renal disease (ESRD).[1,2] This disease is marked by the initial appearance of microalbuminuria.[3] Annual ESRD incidence worldwide has been reported to escalate at an alarming rate, in particular, due to diabetic kidney disease.[4,5]

In addition to other recognized promoters of diabetic nephropathy including chronic hyperglycemia,[6] systemic and intra-renal hypertension,[7] dyslipidemia,[8] smoking,[9] obesity, aging, high degree of insulin resistance, male sex, race, possibly high dietary protein intake[1] and familial clustering of diabetic nephropathy,[10] the effects of genetic polymorphisms cannot be overlooked. Polymorphisms in genes encoding PPAR-γ,[11] eNOS,[12] GLUT-1,[13] aldose reductase,[14] MTHFR,[15] apo-E[16] and components of the renin-angiotensin system (RAS) including angiotensinogen, Ang-II receptor type 1,[17] and particularly, the ACE gene,[18] have been implicated in the pathogenesis of diabetic nephropathy.

ACE mediates regulation of blood volume, arterial pressure, cardiac and vascular function, and electrolyte metabolism. Insertion-deletion (I/D) of a 287-bp fragment at intron 16 of the *ACE* gene results in the genotypes II, ID and DD.[19] Polymorphism was thought to affect ACE-mediated physiological functions[20] and to cause differences in plasma ACE expression levels.[21] Conflicting findings in different populations on the relationship between *ACE* I/D gene polymorphism and diabetic nephropathy led us to investigate *ACE* I/D gene polymorphism as a risk factor for the development of diabetic nephropathy in the multiethnic Malaysian population.

## Materials and Methods

### Subject selection and recruitment

Informed consent was obtained from all subjects with approval granted by the Medical Ethics Committee, prior to sample collection.

Non-diabetic, non-nephropathic, normotensive healthy unrelated control subjects were randomly selected and recruited from local community centers (*n* = 137). Patients enrolled in the study consisted of type 2 diabetes mellitus (T2DM) patients with various stages of nephropathy on follow-up at the outpatient diabetes clinic (*n* = 196) and those undergoing dialysis treatment (*n* = 60). The subjects were initially grouped into four categories based on the urinary albumin–creatinine ratio (ACR): normoalbuminuria (<29 µg/mg creatinine), microalbuminuria (30–299 µg/mg creatinine), proteinuria (>300 µg/mg creatinine) while ESRF subjects were on dialysis.[3] These subjects were further re-grouped into two subgroups using urinary ACR as the cut-off point: Group 1: T2DM without significant nephropathy (ACR ≤ 300 µg/mg creatinine); Group 2: T2DM with significant nephropathy (ACR ≥ 300 µg/mg creatinine), to facilitate statistical analysis according to ethnicity and gender.

### Demographic and clinical characteristics

Anthropometric data such as age and sex were obtained for each subject and relevant clinical parameters including duration (dx) of diabetes, ACR, serum creatinine, GFR, oral glucose tolerance test (OGTT), glycosylated hemoglobin (HbA1C), systolic (SBP) and diastolic blood pressure (DBP), triglyceride (TG) and high density lipoprotein-C (HDL-C) measurement were recorded.

ACR was calculated based on the microalbumin level in the urine sample and serum creatinine level measured by immunoturbidimetric assay[22,23] and modified Jaffe’s kinetic reaction,[24] respectively. The Modified Diet in Renal Disease (MDRD) equation was used to estimate the GFR.[25] OGTT was performed on control subjects following the guidelines described.[26] The HbA_1C_was measured as the ratio of total Hb to HbA_1C_determined by the cyanide-free colorimetric method and immunoturbidimetric assay,[27,28] respectively. At least two readings of SBP and DBP were measured from the subjects who had rested adequately before measurement, using a digital electronic blood pressure (Omron Model M5, Omron Healthcare, Singapore). The measurement of serum TG was based on the kinetic bichromatic enzymatic technique[29] whilst the accelerator selective detergent methodology (Siemens Healthcare Diagnostics, Camberley, UK) was used to measure the HDL-C concentration.

### ACE I/D genotyping

Genomic DNA was isolated using a Wizard^®^Genomic DNA purification kit (Promega, Madison, WI, USA). Triple primer polymerase chain reaction (PCR) and subsequent agarose gel electrophoresis were essentially employed as described.[30] Distinct banding patterns enabled the genotypic determination of the ACE I/D allele as II, ID and DD genotypes. Genotypes were validated by single pass DNA sequencing (Research Biolabs, Singapore) on randomly selected samples. All samples were analyzed in duplicate and were stored at –20°C.

### Data analyses

Genepop DOS version 3.3 Population Genetics Software Package was used to test genetic equilibrium of the *ACE* gene.[31]

Statistical Package for Social Sciences (SPSS) version 14.0 (Chicago, IL, US) was used to perform the statistical analysis. Parametric one-way analysis of variance (ANOVA) test was used to compare means of a quantitative variable between two or more groups when equal variances were assumed, while the non-parametric Kruskal-Wallis test was used when the assumption for equal variances was not met. Levene’s test of homogeneity was used to verify the assumption. Subsequently, Tukey’s HSD and Dunnett’s T3 *post hoc* test were used, respectively, to ensure that the overall probability of declaring any significant differences between all possible pairs of groups is maintained at a significant level of 0.05. Chi-square test was used for association studies between the *ACE* I/D gene polymorphism and disease etiology, ethnicity and gender. The stepwise multinomial logistic regression procedure was used to assess and identify the significant predictors of diabetic nephropathy in our population. P values less than 0.05 were considered significant.

## Results

### Demographic and clinical characteristics

Demographic and clinical characteristics of the subjects are shown in Table 1. All subjects included were characterized according to the nephropathy status. ACR ranges observed between the subgroups were in agreement with the ranges outlined.[3] Similarly, there was no overlap between the ACR categories, thus providing a clear demarcation of subgroups.

**Table 1:** Demographic and clinical characteristics

Parameter	Healthy control	T2DM						
		Normo (N)	Micro (M)	Proteinuria (P)	ESRF (E)
n	137	81	48	67	60
Ethnicity (M:C:I)	63:23:51	23:22:36	20:11:17	31:10:26	38:14:8
Gender (♂/♀)	39/98	31/50	19/29	30/37	30/30
ACR (µg/mg creatinine)	NA	8.01 (2.8–21.7)	97.95 (33.3–241.6)	735.95 (354.2–3062.2)	NA
Age (years)*	45.1 ± 8.8^a^	57.0 ± 10.2	59.8 ± 10.2	62.7 ± 9.5^b^	57.2 ± 9.0
Serum creatinine (µmol/l)†	63.3 ± 13.9^c^	76.3 ± 250.1	103.3 ± 45.6	200.1 ± 113.5	695.0 ± 317.6
GFR (ml/min/1.73 m^2^)†	107.3 ± 22.4^d^	90.0 ± 27.7	67.6 ± 25.3	39.3 ± 27.2	10.7 ± 12.8
OGTT-2hPG (mmol/l)	5.8 ± 0.9	NA	NA	NA	NA
HbA1C (%)†	5.55 ± 0.33^e^	7.71 ± 1.73	8.20 ± 2.24	8.18 ± 2.22	7.49 ± 1.69
Diabetes dx (years)	NA	10.2 ± 7.9	13.1 ± 9.4	16.3 ± 9.4	18.6 ± 6.9
SBP (mm Hg)†	119.6 ± 11.0^f^	136.6 ± 17.4^g^	145.6 ± 25.1	147.7 ± 25.2	162.0 ± 34.1
DBP (mm Hg)†	75.5 ± 7.9^h^	80.9 ± 10.9	80.7 ± 14.9	79.5 ± 11.9	81.6 ± 13.7
TG (mmol/l)†	1.2 ± 0.6^i^	1.4 ± 0.7^j^	1.7 ± 0.9	2.2 ± 2.1	2.2 ± 1.6
HDL (mmol/l)*	1.33 ± 0.32^k^	1.16 ± 0.42	1.13 ± 0.33	1.06 ± 0.37	1.01 ± 0.41

n = number of subjects; M = Malay; C = Chinese; I = Indian; NA = not applicable; All were expressed in mean ± SD except ACR in median (10^th^–90^th^ percentile);

* One-way ANOVA and Tukey *post hoc test*;

† Kruskal-Wallis and Dunnett’s T3 *post hoc test*;

a *P* < 0.05 compared with groups N and E;

b *P* < 0.05 compared with groups N and E;

c *P* < 0.05 compared with groups N and E;

d *P* < 0.05 compared with groups N and E;

e *P* < 0.05 compared with group N and E;

f *P* < 0.05 compared with groups N and E;

g *P* < 0.05 compared with groups P and E;

h *P* < 0.05 compared with groups N and E;

i *P* < 0.05 compared with groups M and E;

j *P* < 0.05 compared with group E;

k *P* < 0.05 compared with groups N and E

Significant mean age and HDL level differences between the groups were observed. The healthy subjects were younger and had higher levels of HDL-C compared to the type 2 diabetic nephropathy subgroups (*P* < 0.05).

We have also observed significant mean rank differences in serum creatinine, GFR, HbA_1C_, SBP, DBP and TG between the groups (*P* < 0.05). The lowest mean serum creatinine concentration was exhibited by the healthy controls, followed by the patient subgroups in an ascending manner, according to the severity of nephropathy. On the other hand, the healthy controls were observed to have the highest mean GFR, followed by normoalbuminuric, microalbuminuric, proteinuric and the ESRF subjects. Also, mean HbA_1C_, SBP, DBP and TG measured among the healthy controls were significantly lower compared to patient subgroups (*P* < 0.05).

### ACE I/D genotyping

**Figure 1:**
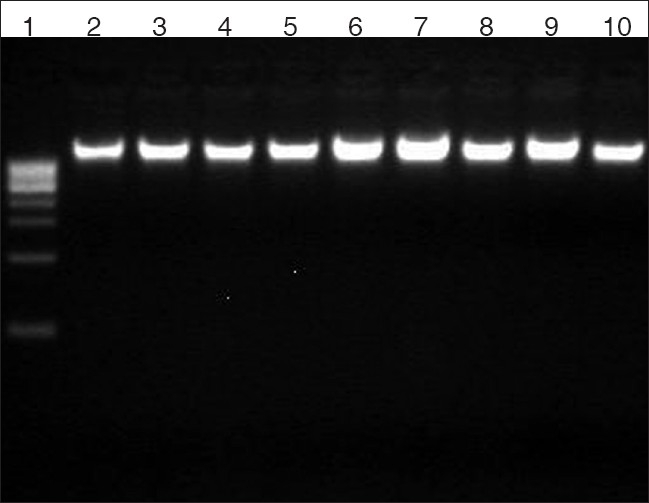
Gel showing DNA extracts (lane 1: 1 kb DNA ladder, lanes 2–10: DNA extracts)

**Figure 2:**
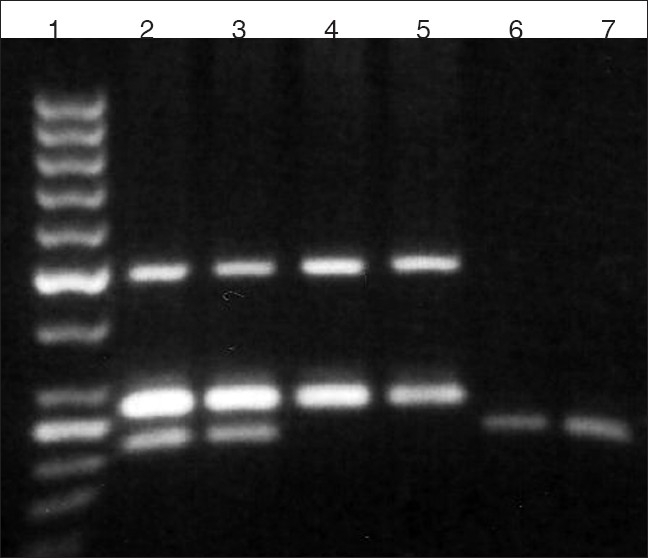
ACE I/D genotype determination under the optimized PCR condition. Band sizes of 495 and 265 bp designated the II genotype, while band sizes of 495, 265 and 210 bp designated the ID genotype and a band size of 210 bp indicates DD genotype (lane 1: 50 bp DNA ladder, lanes 2–3: ID genotype, lanes 4–5: II genotype and lane 6–7: DD genotype)

DNA extraction using fresh blood samples yielded high concentration of DNA [Figure 1]. Typical triple primer PCR results are shown in Figure 2.

### Genetic Susceptibility of ACE gene polymorphism on T2DM with nephropathy

The genotypes and allele frequency of the ACE gene among healthy controls and subjects categorized by the stages of diabetic nephropathy are tabulated in Table 2. The frequencies of ACE I and D allele were consistent with the Hardy-Weinberg equilibrium in all subgroups (*P* > 0.05). Using the chi-square test, we found that there was no significant association between ACE genotype and susceptibility to T2DM (*P* = 0.900). There was also no significant association between ACE genotype and severity of diabetic nephropathy; normoalbuminuria versus microalbuminuria (*P* = 0.085), normoalbuminuria versus macroalbuminuria (*P* = 0.256) and normoalbuminuria versus ESRF (*P* = 0.313).

**Table 2:** Frequencies of ACE genotypes and alleles in healthy controls and T2DM patients with various stages of nephropathy

ACE	Healthy (H) (n = 137)		T2DM (n = 256)							
			Normo (N) (n = 81)		Micro (M) (n = 48)		Macro (P) (n = 67)		ESRF (E) (n = 60)	
	n	Freq.	n	Freq.	n	Freq.	n	Freq.	n	Freq.
Genotype										
II	61	44.5	31	38.3	24	50.0	26	38.8	27	45.0
ID	56	40.9	31	38.3	20	41.7	32	47.8	25	41.7
DD	20	14.6	19	23.5	4	8.3	9	13.4	8	13.3
	*P* value* versus H					1NS†				
			*P* value* versus N		2NS		3NS		4NS	
Allele										
I	178	0.65	93	0.57	68	0.71	84	0.63	79	0.66
D	96	0.35	69	0.43	28	0.29	50	0.37	41	0.34
	*P* value* versus H					1NS†				
			*P* value* versus N		2NS		3NS		4NS	

*n* = number of cases; Freq. = frequency in percentages; NS = not significant;

1 H versus T2DM;

2 N versus M;

3 N versus P;

4 N versus E;

5 Categorical variables, therefore Chi-square was used for the association study;

6 no significant association between *ACE* I/D gene polymorphism and pooled T2DM subjects was found when further demarcated according to ethnicity and gender (*P* > 0.05)

With respect to ACE alleles, Chi-square tests resulted in P values as follows; healthy versus pooled T2DM (*P* = 0.741), normoalbuminuria versus microalbuminuria (*P* = 0.112), normoalbuminuria versus macroalbuminuria (*P* = 0.467) and normoalbuminuria versus ESRF (*P* = 0.235). Since all P values were greater than 0.05, it demonstrates null association between the *ACE* alleles and diabetic nephropathy, irrespective of severity of nephropathy.

### Influence of ethnicity and gender on diabetic nephropathy

Regrouping of the patient subjects using ACR at a cut-off point at 300 µg/mg creatinine resulted in Group1 (*n* = 129) and Group2 (*n* = 127). Table 3 shows the distribution of *ACE* genotype and *ACE* alleles in healthy, T2DM without nephropathy (Group 1) and T2DM with nephropathy (Group 2) subjects based on ethnicity. Malay and Indian ethnic groups showed comparable distribution for the *ACE* genotypic frequency between the groups, demonstrating a null association between *ACE* genotype and diabetic nephropathy. The Chinese ethnic group however, showed a significant difference in the *ACE* genotypic distribution between groups. A greater incidence of ID genotype (66.7%) among the Group 2 subjects was observed. All ethnic groups demonstrated comparable *ACE* allelic distribution, thus demonstrating a null association between *ACE* allele and diabetic nephropathy.

**Table 3:** Ethnicity and diabetic nephropathy

Ethnicity	ACE		Healthy (n = 63)	Group 1 (n = 43)	Group 2 (n = 69)	*P* value*
Malay (n = 175)	Genotype	II	30 (47.6)	22 (51.2)	35 (50.7)	0.931
		ID	26 (41.3)	18 (41.9)	26 (37.7)	
		DD	7 (11.1)	3 (7.0)	8 (11.6)	
	Allele	I	86 (68.3)	62 (72.1)	96 (69.6)	0.914
		D	40 (31.7)	24 (27.9)	42 (30.4)	
Chinese (n = 80)	Genotype	II	13 (56.5)	14 (42.4)	7 (29.2)	0.042
		ID	6 (26.1)	12 (36.4)	16 (66.7)	
		DD	4 (17.4)	7 (21.2)	1 (4.2)	
	Allele	I	32 (69.6)	40 (60.6)	30 (62.5)	0.781
		D	14 (30.4)	26 (39.4)	18 (37.5)	
Indian (n = 138)	Genotype	II	18 (35.3)	19 (35.8)	11 (32.4)	0.903
		ID	24 (47.1)	21 (39.6)	15 (44.1)	
		DD	9 (17.6)	13 (24.5)	8 (23.5)	
	Allele	I	60 (58.8)	59 (55.7)	37 (54.4)	0.866
		D	42 (41.2)	47 (44.3)	31 (45.6)	

Group 1, T2DM without nephropathy; Group 2, T2DM with nephropathy;

* Categorical variables, therefore Chi-square was used for the association study; n = number of cases; percentages in parentheses

Table 4 shows the influence of gender on *ACE* genotypic and allelic distribution in healthy, Group 1 and Group 2 subjects. The distribution of *ACE* genotypes and alleles was not different between the groups for both male and female categories.

**Table 4:** Gender and diabetic nephropathy

Gender	ACE		Healthy (n = 39)	Group 1 (n = 50)	Group 2 (n = 60)	*P* value*
Male (n = 149)	Genotype	II	18 (46.2)	28 (56.0)	22 (36.7)	0.176
		ID	14 (35.9)	15 (30.0)	31(51.7)	
		DD	7 (17.9)	7 (14.0)	7 (11.7)	
	Allele	I	50 (64.1)	71 (71.0)	75 (62.5)	0.914
		D	28 (35.9)	29 (29.0)	45 (37.5)	
Female (n = 244)	Genotype	II	43 (43.9)	27 (34.2)	31 (46.3)	0.506
		ID	42 (42.9)	36 (45.6)	26 (38.8)	
		DD	13 (13.3)	16 (20.3)	10 (14.9)	
	Allele	I	128 (64.0)	90 (57.0)	88 (65.7)	0.781
		D	68 (34.7)	68 (43.0)	46 (34.3)	

Group 1, T2DM without nephropathy; Group 2, T2DM with nephropathy;

* Categorical variables, therefore Chi-square was used for the association study; n = number of cases; percentages in parentheses

### Potential predictors of diabetic nephropathy

All potential risk factors for diabetic nephropathy including age, ethnicity, blood pressure, HDL, triglyceride, gender, *ACE* genotype and HbA_1C_were assessed by stepwise logistic regression procedure. T2DM patients with proteinuria and ESRF were evaluated with reference to healthy controls by this model. The results of fitting the stepwise logistic regression model for diabetic nephropathy are presented in Table 5. Factors that are independently associated with diabetic nephropathy are age, SBP, HDL, and HbA_1C_, in that order.

**Table 5:** Stepwise logistic regression model for diabetic nephropathy

Effect	Likelihood ratio test		
	Chi-square	df	*P* value
Age	26.076	2	0.000
SBP	43.515	2	0.000
HDL	8.092	2	0.017
HbA1C	87.585	2	0.000

df = Degree of freedom. Factors that are not associated with diabetic nephropathy are not shown

## Discussion

The true genetic susceptibility of ACE I/D gene polymorphism on disease pathogenesis remains unelucidated due to contrasting outcomes worldwide. Table 6 shows the various findings linking the influence of ACE I/D gene polymorphism to diabetic nephropathy. Publication bias, differences in study design, patient selection, case and control definition, effect of disease progression or regression,[32] mistyping of genotypes, small sample size and particularly, ethnic differences were thought to be the causes of these outcomes.[33]

**Table 6:** Association studies between ACE I/D gene polymorphism and diabetic nephropathy worldwide

Population	Total samples	Case definition*	Control definition	Result
Malaysian	240	T2DM	Healthy control	Negative
Malay		DN	WN	Negative
Malaysian	138	T2DM	Healthy control	Negative
Chinese		DN	WN	Negative
Malaysian	189	T2DM	Healthy control	Negative
Indian		DN	WN	Negative
Caucasian-American[38]	509	DN	Healthy control	NA
			WN	Positive
Caucasian-Polish[39]	108	DN	Healthy control	NA
			WN	Negative
Taiwanese[40]	599	T2DM	Healthy control	Positive
		DN	WN	Positive
Japanese[41]	467	T2DM	Healthy control	Negative
		DN	WN	Positive
Korean[42]	191	T2DM	Healthy control	NA
		DN	WN	Positive
Chinese[43]	168	DN	Healthy control	NA
			WN	Negative
				
Chinese[44]	1281	DN	Healthy control	NA
			WN	Positive
South Indian[18]	460	DN	Healthy control	Negative
			WN	Positive
South Indian[45]	109	DN	Healthy control	NA
			WN	Positive
North Indian[46]	117	DN	Healthy control	Negative
			WN	NA
Turkish[47]	377	DN	Healthy control	NA
			WN	Negative
Turkish[48]	112	T2DM	Healthy control	Positive
		DN		Negative

* Case and control definition for diabetic nephropathy (DN) and T2DM without nephropathy (WN), respectively, may be different between studies. WN = T2DM subjects without nephropathy; NA = not applicable

A number of meta-analyses have been conducted to clarify the role of ethnicity on the association of *ACE* gene polymorphism with diabetic nephropathy. Kunz *et al*.,[34] failed to demonstrate a conclusive correlation in his meta-analysis due to methodological limitations, while Tarnow *et al*.,[35] supported the association of *ACE* I/D gene polymorphism with diabetic nephropathy only among Northeast Asians. In contrast, Fujisawa *et al*.,[36] concluded that *ACE* I/D gene polymorphism was a universal risk factor for diabetic nephropathy. However, Ng *et al*.,[37] presented a comprehensive report based on a larger sample size (*n* = 14,727) supporting the genetic association of *ACE* I/D polymorphism with diabetic nephropathy among Asians.

In the present study, we investigated the possible association of *ACE* I/D gene polymorphism with diabetic nephropathy among Malaysians and took advantage of the presence of the three different ethnic groups, namely Malay, Chinese and Indian, in Malaysia. We observed that a) *ACE* I/D gene polymorphism was significantly associated with neither T2DM nor diabetic nephropathy, b) ethnicity did not alter the null influence of *ACE* I/D gene polymorphism on diabetic nephropathy except among the Chinese T2DM subjects with nephropathy and c) gender-specific associations were not present between the *ACE* genotype and diabetic nephropathy.

Assuming that the influence of *ACE* I/D gene polymorphism on disease pathogenesis is ethnicity/population specific, it is not surprising that we observed a null association between *ACE* I/D gene polymorphism and diabetic nephropathy for the healthy control and patient groups which comprised three different ethnic groups [Table 3]. In contrast, Ramachandran *et al*.,[49] reported a positive association between *ACE* I/D gene polymorphism with diabetes and hypertension among Malaysian subjects.

Demarcation of subjects according to ethnicity shows that the Malay, Chinese and Indian ethnic groups with both T2DM and diabetic nephropathy were not independently associated with *ACE* I/D gene polymorphism, disputing the finding of Ng *et al*.[37] To our knowledge, there have not been any previous studies in the Malay ethnic group on the association between the *ACE* gene and diabetic nephropathy. Sasongko *et al*.,[50] conducted a study on the Javanese-Indonesians demonstrating a negative association between *ACE* gene polymorphism, however, with idiopathic nephrotic syndrome. Therefore, our current study provides a novel finding demonstrating a null association between *ACE* I/D gene polymorphism and diabetic nephropathy, with regard to the Malay ethnic group.

Our findings among the Indian ethnic group were similar to that of a previous study by Kumar *et al*.,[46] but were not in agreement with that of Viswanathan *et al*.,[45] even though both the latter studies were conducted on Asian Indians. Later, however, Movvaa *et al*.,[18] resolved the dispute between the latter two studies using a larger sample size and appropriate case–control definition, hence ascertaining the D alleles as a risk factor for renal complication in T2DM subjects among Asian Indians. Similarly, the discrepancies found among the Indian ethnic group in this study could most likely be due to errors in the technical/methodological aspects rather than real genetic differences.[51]

Our observation with regard to the Chinese ethnic group is in contrast with the findings on Southern Chinese and other Far East Chinese populations, which demonstrated strong evidence of the deleterious effect of the D allele in the etiology of T2DM[40,52,53] and diabetic nephropathy.[40–42,44] Nevertheless, our findings are in agreement with those of Wong *et al*.[43] who investigated the prognostic effect of an *ACE* I/D gene polymorphism in the diabetic nephropathy on Chinese population in Hong Kong, China. Over the years, allelic frequencies’ differences with lower D allele and higher I allele frequencies among Caucasians have been used as a reason for the conflicting outcomes compared to other Asians. Unfortunately, in this case, the fact is that Malaysian Chinese and other Far East populations shared a remarkable similarity in terms of *ACE* I/D allelic distribution,[30] so this argument may not apply. A more stringent case definition of diabetic nephropathy and a larger sample size to elucidate the association of *ACE* I/D gene polymorphism on diabetic nephropathy in the Chinese ethnic group in Malaysia may be necessary.

Interestingly, we also found an independent association between ID genotype and diabetic nephropathy among the Chinese when patients were further subgrouped according to the severity of nephropathy. Although the ID genotype is generally the most frequent type of *ACE* genotype,[54] the plausibility of ID genotype as a potential predictor for diabetic nephropathy is highly unlikely. The D allele was thought to behave as a recessive trait, requiring the presence of two alleles to contribute to the progression of ESRF.[42] In addition, the II, ID and DD genotypes were previously demonstrated to display lowest, intermediate and highest levels of serum ACE levels, respectively.[19] In line with this, the DD genotype has always been held accountable for exerting its deleterious effects on various disease pathogenesis including diabetic nephropathy.

We did not observe any gender-dependent association between the *ACE* I/D gene polymorphism and T2DM or diabetic nephropathy. Although an increase in D allele frequencies was observed among the females, it was not significant enough to alter the genotypic presentation that was thought to be a predisposing factor for the progression of renal disease. The influence of gender on the deterioration of renal function remains controversial. Many have suggested that decline in renal function of patients with chronic renal disease is more rapid in men than in women.[55] The receptor-mediated effects of sex hormone estrogen was thought to exert potent antioxidant actions contributing to renoprotective effects in females. In contrast, other reports point to females being predisposed to diabetic renal disease.[56]

We conclude that in this study the role of *ACE* I/D gene polymorphism susceptibility to both T2DM and/or diabetic nephropathy was not demonstrated. Factors including ethnicity and gender did not alter the null influences of *ACE* I/D gene polymorphism on disease patterns in our population. Although statistically sufficient sample sizes were used in the present study, it would be beneficial to extend the sample size to confirm and validate our present findings. In addition, profiling of polymorphic genes encoding for components of the RAS might confer relevant insight into genetic predisposition to diabetic nephropathy.
